# The Metal-Dependent Regulators FurA and FurB from *Mycobacterium Tuberculosis*

**DOI:** 10.3390/ijms9081548

**Published:** 2008-08-28

**Authors:** Debora Lucarelli, Michael L. Vasil, Wolfram Meyer-Klaucke, Ehmke Pohl

**Affiliations:** 1 European Molecular Biology Laboratory, Hamburg Outstation, Notkestr. 85, D-22603 Hamburg, Germany. E-Mails: Debora.Lucarelli@cancer.org.uk (D. L.); wolfram@embl-hamburg.de (W. M-K.); 2 Department of Microbiology, University of Colorado, Denver Anschutz Medical Campus, Aurora, CO 80045, USA. E-Mail: Mike.Vasil@UCHSC.edu (M. V.); 3 Department of Chemistry & School of Biological and Biomedical Sciences, Durham University, Durham DH1 3LE, UK

**Keywords:** Metal uptake, regulator, ferric, zinc, Mycobacterium tuberculosis

## Abstract

The ferric uptake regulators (Fur) form a large family of bacterial metal-activated DNA-binding proteins that control a diverse set of genes at the transcriptional level. *Mycobacterium tuberculosis*, the causative agent of tuberculosis, expresses two members of the Fur family, designated FurA and FurB. Although both belong to the same family, they share only approximately 25% sequence identity and as a consequence, they differ significantly in some of their key biological functions. FurA appears to be a specialized iron-dependent regulator that controls the *katG* gene, which encodes for a catalase-peroxidase involved in the response of *M. tuberculosis* to oxidative stress. KatG is also the key mycobacterial enzyme responsible for the activation of the first-line tuberculosis drug Isoniazid. FurB in contrast requires Zn^2+^ rather than Fe^2+^, to bind to its target sequence in regulated genes, which include those involved in Zn^2+^-homeostasis. Recent biochemical, crystallographic and spectroscopic data have now shed light on the activation and metal discrimination mechanisms in this protein family.

## 1. Introduction

*Mycobacterium tuberculosis,* a Gram-positive bacteria and a widespread human pathogen, resides mainly in alveolar macrophages within the lungs of infected individuals. *M. tuberculosis* has infected up to one third of the world's population but only a small proportion manifests the disease, tuberculosis (TB). TB is most often associated with poor socio-economic conditions and/or co-infections such as HIV. In 2006, it was estimated that there were approximately 9.2 million new cases of TB, with a death toll of more than 1.5 million people [1]. Even though efficacious short-course chemotherapy (DOTS) and the Bacille Guerin-Calmette (BGC) vaccine are now available for a large proportion of the populations of developed countries, the threat remains, as the incidence of multi-drug resistance is steadily increasing in *M. tuberculosis* [2].

Like all human pathogens, *M. tuberculosis* must contend with limited metal availability in order to survive in the human body. Iron and zinc are essential trace elements, however, both are toxic at elevated concentrations [3, 4]. As the sequestration of iron is part of the non-specific mammalian immune response metal uptake and regulation of metal ion concentrations are central to host-pathogen interactions [5]. Metal ion homeostasis in prokaryotes is generally maintained at the level of transcription by various metal dependent regulator proteins [3, 5, 6]. The *M. tuberculosis* genome contains genes encoding for four of these regulators belonging to two different families [7]. IdeR (for Iron-dependent Regulator) and SirR (for staphyloccocal iron regulatory repressor) belong to the Diphtheria toxin Repressor (DtxR) family. FurA and FurB are members of the second, the ferric uptake regulator (Fur) family.

IdeR is an essential protein in *M. tuberculosis* and plays the central role as an iron-dependent regulator which controls a large cohort of genes encoding proteins required for iron-uptake and storage [8, 9]. A series of crystal structures of holo-IdeR and IdeR in complex with DNA have shown that this protein undergoes a conformational change upon Fe^2+^-binding and most likely binds its target as a double dimer [10–12]. SirR has originally been identified in *Staphylococcus epidermidis* [13] as a 25 kDa protein with approximately 33% sequence identity to IdeR. In *S. epidermidis* SirR (SirR_se_) controls the three-gene operon *sitABC* encoding for an ABC transporter system that has been suggested to be involved in iron-uptake [13, 14]. Furthermore a pseudo-palindromic region in the operator/promoter sequence of *sitABC* has been shown to be specifically recognized by Fe^2+^- or Mn^2+^-activated SirR_se_ [13]. The biological role of the SirR homologue in *M. tuberculosis*, however, has yet to be determined.

Fur was originally identified in *E. coli* where it acts as global regulator influencing the expression of close to 100 genes [15, 16]. Members of the Fur family affect the expression of an array of genes, not only associated with iron acquisition and storage, but also those involved in intermediary metabolism as well as responses to acidic environments and oxidative stress. Moreover, they directly or indirectly control the expression of a variety of genes associated with virulence in Gram-positive and negative bacteria [6, 16–19]. In the initial working model it was suggested that Fur is activated by ferric iron and then binds to its operator, a 19 base pair pseudo-palindrome called Fur-Box [16, 20]. Binding to the operator region of a regulated gene blocks access of the RNA-polymerase thereby repressing the transcription of the downstream gene(s). More recent studies, however, found that the regulation exerted by members of the Fur family is far more intricate than initially perceived. Several homologues, including Fur from *E. coli* (Fur_ec_) contain at least one tightly bound Zn^2+^-ion that is believed to serve a structural rather than a regulatory role [21–23]. Moreover, certain members of the Fur family are activated by divalent transition metals other than Fe^2+^, which include zinc, nickel and manganese. Accordingly, such members have now been named Zur [24], Nur [25] and Mur [26] which connotes that these family members are capable of selectively binding to a specific divalent transition metal other than Fe^2+^ to activate their DNA binding function. Furthermore, Fur can also act as a positive regulatory factor, rather than as a classical repressor. While different mechanisms for this type of regulation have been proposed, in a number of bacterial pathogens, Fur homologues directly repress the expression of non-coding small RNAs named RHyhB in *E. coli* [27] and *V. cholerae* [28]*,* PrrF in *P. aeruginosa* [29] and NrrF in *Neisseria meningitidis* [30] that in turn repress the expression of certain genes. Under iron-starvation conditions RyhB is rapidly expressed and stabilized by the RNA-chaperone Hfq. The single-stranded RNA in the RNA-Hfq complex then pairs with the complementary messenger RNA which becomes susceptible to rapid degradation [31].

## 2. Discussion

### 2.1. Biological role of FurA and FurB in M. tuberculosis

**Figure 1:**
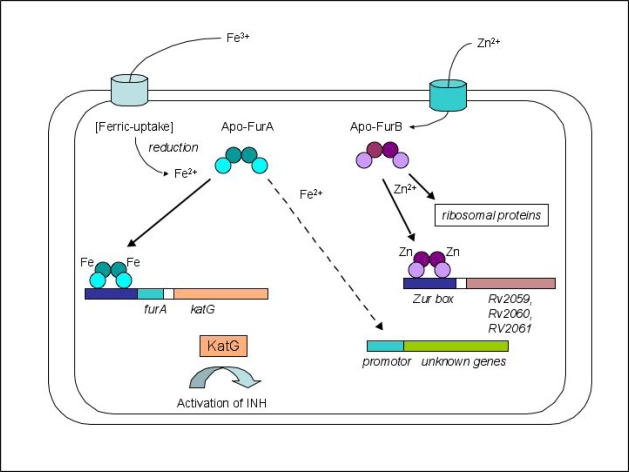
Schematic representation of the regulatory functions of *M. tuberculosis* FurA and FurB, respectively. Full arrows refer to proven repression, dashed arrows refer to proposed activation pathways. Note, that the structural Zn^2+^-site present in both FurA and FurB has been omitted for clarity.

**Figure 2:**
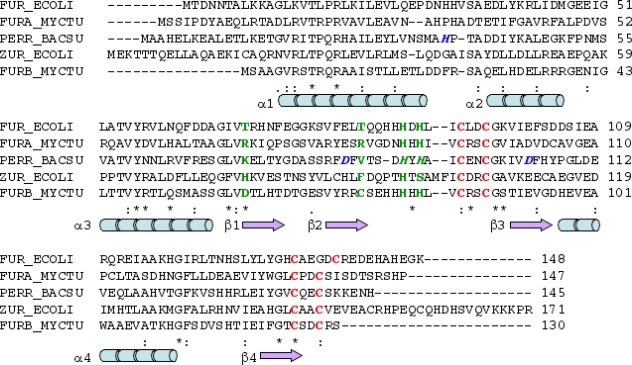
Sequence alignment of Fur_ec_ with FurA, PerR_bs_ together with Zur_ec_ and FurB (performed with ClustalW2 [45]). Residues of the putative regulatory binding site identified in the crystal structure of FurB are depicted in green, residues of the putative structural binding site are red [43]. The proposed binding residues for the second, regulator binding site in PerR_bs_ His37, Asp85, His91, His 93 and Asp104 are shown in blue [46]. Secondary structure assignment is based on the FurB crystal structure: blue rods refers to α-helix, and violet arrows to β-strands. The last line shows sequence conservation: '*' denote conserved residues, '.' and ':' indicate similar residues in the alignment.

The *furA* gene is located immediately upstream of the *katG* gene encoding a catalase-peroxidase. KatG is involved in the oxidative stress response and a significant virulence factor of *M. tuberculosis* [32]. FurA is co-expressed along with KatG and it auto-represses its expression by binding to a unique sequence upstream of the *furA* gene [33]. Although there are indications that this protein is also involved in the regulation of other genes [32], the biological role of FurA, in contrast to the role of most members of the family appears to be more specialized (Figure 1). FurA could represent the metal-dependent peroxide sensor similar to the peroxide regulon repressor PerR that has been extensively characterized in *B. subtilis* [34]. Both proteins share a sequence identity of approximately 28% (Figure 2). PerR_bs_ adopts a similar fold to Fur_pa_ and contains one structural Zn^2+^- and one regulatory Fe^2+^/Mn^2+^-binding site [35]. Once activated and bound to its target DNA-sequence the regulator senses oxidative stress by iron-catalyzed histidine oxidation that leads to the loss of DNA-binding activity [36]. FurA is of particular biomedical importance as it controls a protein central to the contemporary TB therapy. One of the more efficacious therapeutics for this disease is Isoniazid (isonicotinic acid hydrazide = INH), which is able to traverse the complex lipid membrane of *M. tuberculosis*, entirely by passive diffusion. While unmodified INH is not toxic to the pathogen it becomes activated by the mycobacterial catalase KatG, which modifies INH into a range of reactive intermediates including NAD^+^ and NADP^+^ adducts. These then act as potent inhibitors of the NADPH-dependent enoyl acyl carrier protein reductase (InhA) of the fatty acid synthase type II [37, 38]. InhA is an essential enzyme in the mycolic acid synthesis [39]. INH-resistant *M. tuberculosis* strains isolated from patients predominantly have mutations in the *katG* gene leading to a catalase-peroxidase with reduced or abolished catalytic activity [38]. In addition, mutations were also found in the *inhA* gene as well as various other genes including some in the *furA* gene [38, 40]. FurA has attracted increased interest as a potentially novel drug target for the development of inhibitors that could enhance KatG levels possibly boosting INH potency.

The biological role of *M. tuberculosis* FurB has only recently been examined. Elegant biological and biochemical studies [41, 42] as well as biophysical and structural analyses [43] strongly suggested that FurB represents the authentic Zinc uptake regulator (Zur) in *M. tuberculosis* (Figure 1). The *furB* gene is co-transcribed with its upstream gene (Rv2358), which encodes another zinc-dependent regulator. In the absence of Zn^2+^ the gene product of Rv2359 represses the expression of both genes [41]. FurB is responsible for repressing at least 32 genes a number of which have been implicated with zinc homeostasis. For instance, the triplet gene cluster Rv2059-Rv2061c encodes for two components of an ABC-transport system, Rv2059 shows homology with the TroA superfamily, Rv2060 is similar to a membrane protein part of an ABC-type Mn^2+^/Zn^2+^ transport system [42, 43]. However, since the two reading frames of Rv2059 and 2060 overlap it is not clear if functional proteins are expressed from these genes [42]. In addition, FurB controls five genes encoding ribosomal proteins three of which containing a putative zinc-binding motif. Based on comparative genomics data these ribosomal proteins have also been suggested to be involved in zinc homeostasis [44]. While FurB plays an important biological role as the genuine zinc-dependent repressor no phenotype difference was observed between wild-type and a *M. tuberculosis* strain in which the *zur* gene was deleted [42].

Although FurA and FurB belong to the same protein family they only share a sequence identity of approximately 25%. Thus, while it is likely that they have a similar overall structure, certain key elements in their respective architectures are likely to differ considerably. For instance, some of the distinct structural details discriminate in divalent metal binding while others are critical to the recognition of DNA sequence motifs in the operator of regulated genes.

### 2.2. Crystal structure of FurB

**Figure 3:**
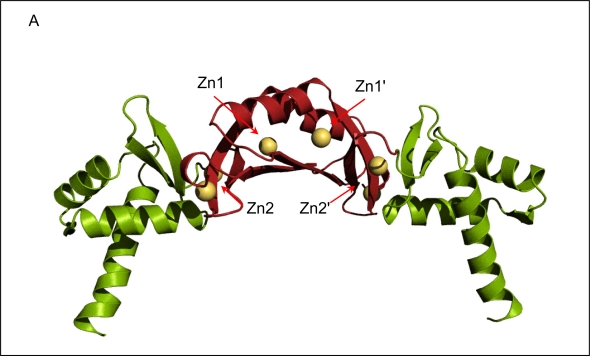

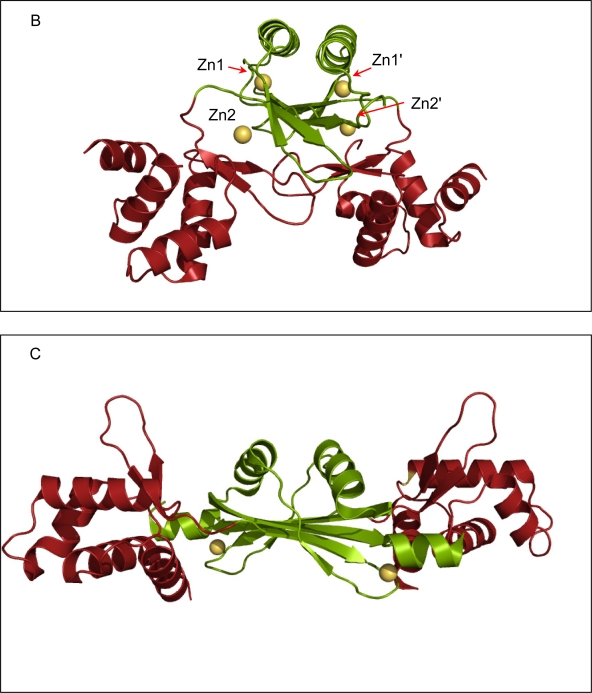
Ribbon diagrams of the crystal structure of Fur homologues. All three proteins are dimers with the N-terminal DNA-binding domain depicted in green and the C-terminal dimerization domain shown in red. All Zn^2+^-ions are shown as golden spheres. (a) FurB in complex with Zn^2+^ solved at 2.7 Å resolution, the putative regulatory sites are labeled Zn1 and Zn1', the structural sites are labeled Zn2 and Zn2', respectively. The labels for the third site were omitted for clarity [43]. (b)Fur_pa_ complexed with Zn^2+^ [47]. (c)apo-PerR_bs_ with its putative structural Zn^2+^-site [46]. All Figures were produced with PyMol [50].

The crystal structure of FurB in complex with Zn^2+^ determined at a resolution of 2.7 Å revealed the familiar two-domain structure with the N-terminal DNA-binding domain composed of a three-helix bundle followed by a short antiparallel β-sheet, and the C-terminal metal-binding and dimerization domain [43]. Although the overall fold is similar to the first crystal structure of another member of the Fur-family (Fur from *P. aeruginosa*, Fur_pa_ [47]) there are noteworthy differences in domain orientation as well as metal binding sites. Overall, the individual domains of FurB are very similar with the exception that the DNA-binding domain of FurB lacks the N-terminal helix that is present in Fur_pa_ and Fur_ec_ [48]. However, the relative orientations of their two domains are very different (Figure 3a-c). The FurB homodimer adopts a much more open conformation where the DNA-binding domains are further separated and the DNA-recognition helices (helix 3, residues 45–58 in FurB in Figure 2) are almost collinear to each other. While the amino-acid sequence of the DNA binding helix is well conserved within the Fur family there are sufficient differences to enable DNA sequence specific recognition. The mobility of DNA-binding domains with respect to the dimer interface is also highlighted by the crystal structure of apo-PerR_bs_ which shows a similar fold but a distinctively different domain orientation [46]. It should be noted that such domain motions can also be caused by packing effects as have been observed previously in the crystal structures of apo- and holo-DtxR [49].

### 2.3. Metal binding sites and metal selectivity

**Figure 4:**
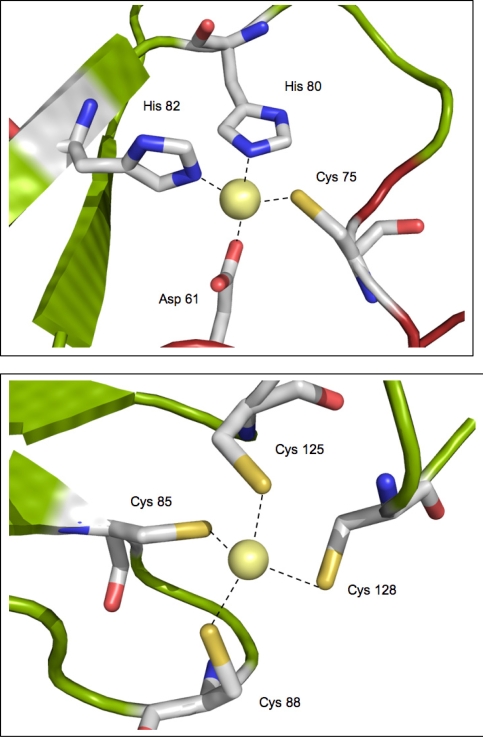
Close-up of the two metal binding sites in the crystal structure of FurB: (a) putative regulatory Zn^2+^-site. (b) putative structural Zn^2+^-site. The residue numbers refer to the sequence given in Figure 1.

Three Zn^2+^-binding sites were identified in the crystal structure of FurB, two of which were further characterized by EXAFS measurements in solution. The biological role of the third Zn^2+^-site is not clear and it seems likely that this site may represent a crystallization artifact. The first metal site depicted in Figure 4a, revealed a Zn^2+^-ion tetrahedrally coordinated by Asp61 (corresponding to Thr69 in Fur_ec_), Cys75 (Thr83), His80 and His82, respectively (His88 and His90). Further spectroscopiC analysis showed that this site can be readily exchanged against Co^2+^-ions with the same coordination sphere [43], hence suggesting it represents the lower-affinity regulatory site used to switch the DNA-binding capability of FurB to *on*. It should be noted that this site corresponds roughly to the originally denoted putative structural site in Fur_pa_ [47]. However, more recent data indicate that this assignment based on crystallographic and spectroscopic data may not represent the biologically relevant state [18].

The sequence comparison shows that the residues of this site depicted in green in the sequence alignment (Figure 2) are only partially conserved, presumably because the different members of the Fur family have evolved to recognize different metals, possibly at different concentrations, thereby fine-tuning the regulatory networks in each organism. It is noteworthy that both Asp 61 and Cys75 in FurB are not conserved in FurA, rather they are changed to arginine residues, which cannot serve as ligands for divalent transition metals. Thus, it is likely that in FurA the framework of the overall Fur fold provides a chemically different metal environment better suited for Fe^2+^ than Zn^2+^. Compared to zinc ions, ferric iron prefers octahedral coordination with O,N ligands rather than tetrahedral coordination with S-containing ligands [51]. Considering that the FurA sequence shows a number of histidine and aspartic acid residues in the vicinity a preferred Fe^2+^-binding site can be realized without major structural changes. The residues that have been suggested to constitute the second, regulatory binding site in PerR_bs_ (depicted in italic and blue/green in Figure 2) are in fact highly conserved in FurA (His37, Asp85, His91,His93 and Asp104, numbering according to PerR_bs_). Thus, it is possible, that FurA employs a similar mechanism to function as a peroxide sensor in *M. tuberculosis*.

The second metal binding site in FurB depicted in Figure 4b represents a regular tetrahedral coordination of the four cysteine residues Cys85, Cys88, Cys125 and Cys128. ZnS4 clusters in this arrangement have been observed in various proteins and are typical for structural Zn^2+^-sites [51, 52]. The notion of a structural Zn^2+^-site that ties the N-terminus to the core of the dimer was further supported by a series of experiments [43]. First of all it was not possible to completely remove the Zn^2+^ from FurB without precipitating the protein sample presumably due to (partial) unfolding. Secondly, microPIXE analysis [53] showed approximately one zinc atom per monomer and thirdly, the same tetrahedral ZnS_4_ environment was observed in EXAFS studies performed using a protein solution that had not been incubated with any additional metal. Moreover, a similar structural Zn^2+^ site was also revealed in the recent crystal structure of apo-PerR_bs_ [46]. Even though the sequence alignment appears to indicate that all cysteine resides in this cluster are highly conserved between Fur_ec_, FurA, Zur_ec_ and FurB (depicted in red in Figure 2) it should be noted that a series of mutational and spectroscopic experiments point to slightly different Zn^2+^ surroundings for the structural sites ranging from Zn(S)_2_(O/N)_2_ in Fur_ec_ to Zn(S)_3_(O/N) in Zur_ec_ [21, 54]. This metal site may be structurally less well conserved than deduced from sequence alone. However, preliminary EXAFS analysis on apo-FurA confirms a single Zn^2+^-ion tightly bound in a structural binding site. (Lucarelli, Pohl, Meyer-Klaucke, unpublished data). It is therefore likely that FurA and FurB share the same structural Zn^2+^-spot.

## 3. Conclusions

The past several years have seen significant progress in understanding the control of gene expression by metal dependent transcriptional regulators in *M. tuberculosis* and other bacterial pathogens. FurB has been identified as the Zinc uptake regulator (Zur) [42], while FurA controls the production of the catalase-peroxidase KatG, an essential enzyme in INH therapy of TB treatment [32]. FurA could thus represent the functional homologue of the peroxide sensor PerR. In general the mechanisms governing gene repression are reasonably well understood. By contrast, the full cohort of genes (activated and repressed) controlled by these Fur family members, and other metal-dependent regulatory proteins have not yet been identified, nor have the mechanisms associated with their activation been fully elucidated. Although metal binding and activation mechanism have now been examined in greater detail for a significant number of Fur family members, including FurA and FurB, their exact mode of DNA-binding and in particular the structural basis of specific target recognition, remains unknown. Further knowledge about the architecture and function of Fur-operator complexes will certainly enhance future structurally driven drug design efforts.
